# Improvement of Mouse Spermatozoa Freezing at −80°C With Ascorbic Acid 2‐Glucoside at Thawing Phase

**DOI:** 10.1111/andr.70228

**Published:** 2026-04-12

**Authors:** Alessia Paradiso, Renata Paoletti, Simone Cassetti, Nicola Bernabò, Barbara Barboni, Marcello Raspa, Ferdinando Scavizzi

**Affiliations:** ^1^ Department of Bioscience and Technology for Food Agriculture and Environment University of Teramo Teramo Italy; ^2^ Plaisant S.r.L Rome Italy; ^3^ Tecniplast S.p.A, Buguggiate Varese Italy; ^4^ Institute of Biochemistry and Cell Biology (CNR‐IBBC/EMMA/Infrafrontier/IMPC) National Research Council Monterotondo Scalo Rome Italy

**Keywords:** antioxidant, cryopreservation, mouse, reproductive technology, spermatozoa, ultra‐low freezer

## Abstract

**Background:**

Sperm cryopreservation is the most common method to maintain a great number of mutant mouse lines. However, the use of liquid nitrogen (LN_2_) for freezing presents considerable problems in terms of cost, safety, and accessibility. For this reason, the storage of semen samples at ‐80° in ultra‐freezers has been increasingly used in recent years, with evident advantages: it does not require dedicated space, it is cheaper, and it is available even in small laboratories.

**Objectives:**

In this work, freezing at ‐80°C is tested in combination with antioxidant, aimed at reducing membrane damage and oxidative stress.

**Materials and Methods:**

Wildtype mouse line sperm were cryopreserved using the CPM method directly at ‐80°C. The effect of ascorbic acid 2‐O‐alpha‐glucoside (AA2G) spermatozoa treatment, during thawing, was evaluated through a qualitative (motility, vitality, and acrosomal reaction using SCA System) and functional analysis (IVF and embryo development), after 1, 6, and 12 months.

**Results:**

Sperm motility and velocity parameters showed no significant differences between treated and control groups across both B6N and B6J strains over 12 months, though overall motility declined with time. Antioxidant treatment (AA2G) significantly improved sperm viability and in vitro fertilization (IVF) outcomes in both strains, particularly B6N, without affecting acrosome reaction rates or blastocyst development.

**Discussion:**

Sperm cryopreservation at ‐80°C, while cost‐effective, induces strain‐dependent damage affecting sperm viability and fertility, particularly in B6J mice. Supplementation with the antioxidant AA2G during capacitation significantly improved post‐thaw sperm survival and in vitro fertilization outcomes in both B6N and B6J strains, supporting its use in optimizing cryopreservation protocols without liquid nitrogen.

**Conclusion:**

These results may help to design new protocols or optimize those already validated by promoting the use of antioxidants in freezing at ‐80° as they have a significant effect on reducing oxidative stress, making spermatozoa qualitatively comparable to freezing in LN2.

## 1 Introduction

Since the completion of the human and mouse genome projects in 2001 and 2002 respectively, significant advancements have been made in the creation of genetically altered (GA) mouse lines through large‐scale gene knockout programs [1, 2]. In order to optimize the utilization of these resources, it is imperative that comprehensive collections of mouse models are cryopreserved and made readily accessible to researchers globally [3]. The establishment of collaborative research infrastructures, such as the International Mouse Phenotyping Consortium (IMPC) [4, 5], has further amplified the demand for these novel strains, while innovative techniques like CRISPR/Cas9 have expedited the generation of GA mice [6]. In order to align with ethical principles such as the 3Rs (Replacement, Reduction, and Refinement) and adhere to legal standards, the dissemination of mouse strains in cryopreserved forms, such as embryos or spermatozoa, has become a priority [7].

The process of cryopreservation, first established for laboratory mice in 1972, has since been refined to facilitate the efficient storage and recovery of genetic material [8, 9, 10]. The preservation of laboratory mouse genomes is a cornerstone of biomedical and translational research, given the genetic and physiological similarities between mice and humans [11]. Cryopreservation has been the key method to archive and distribute genetic resources. Embryo freezing has been the traditional approach for securing mouse strains, but sperm freezing has gained traction in recent years due to its cost‐effectiveness, simplicity, and suitability for large‐scale storage and transport [12, 13]. Nevertheless, the process of sperm freezing still relies on liquid nitrogen (LN_2_), which introduces significant risks, considerable expense, and logistical challenges [11].

Recent advances have explored alternative methods for freezing and storing mouse sperm, focusing on ultra‐low freezers at ‐80°C [14]. This approach has demonstrated potential in offering a safer and more cost‐effective solution while maintaining sperm viability and functionality. Our research group has previously demonstrated that mouse sperm can be stored at ‐80°C for both brief periods [15] and up to 5 years, without compromising its capacity for fertilization [11].

Another critical issue in the domain of sperm cryopreservation pertains to the oxidative stress that is instigated during the freezing and thawing processes. The generation of excessive reactive oxygen species (ROS) during these processes has been demonstrated to result in the damage of sperm DNA, the reduction of motility, and the impairment of fertilization capability. Antioxidants have been employed extensively to mitigate these effects, with ascorbic acid (vitamin C) being one of the most effective due to its potent ROS‐scavenging properties [16]. Despite this, ascorbic acid's instability and rapid degradation limit its application in cryopreservation.

We, previously demonstrated that Ascorbyl glucoside (AA2G), a water‐soluble derivative of vitamin C (ascorbic acid) that is combined with glucose, was more stable and less likely to degrade when exposed to air, light, and heat, compared to pure vitamin C, making it a good candidate for improving sperm quality and IVF success after storage in liquid nitrogen [16]. AA2G functions as a powerful antioxidant, protecting cells from oxidative stress and enhancing their overall viability. By neutralizing harmful reactive oxygen species (ROS), it helps prevent cellular damage to proteins, lipids, and DNA. Additionally, it plays a crucial role in stabilizing other antioxidants, such as glutathione and vitamin E, ensuring a balanced redox environment.

Its stability allows for a slow, sustained release of ascorbic acid, preventing degradation of key nutrients in the culture medium. Furthermore, it helps shield cells from light‐induced oxidative stress, preserving the integrity of the culture over time.

We also reported that C57BL/6N spermatozoa can be efficiently frozen and maintained at ‐80°C, without use of LN_2_ at any stage, for 12 months [14]. Now, this study evaluates the effects of AA2G on key sperm parameters, including motility, viability, acrosomal reaction, and fertilization capacity, using inbred mouse strains C57BL/6J and C57BL/6N after 1, 6, and 12 months at ‐80°C. To tackle the challenges outlined earlier, this study explores the application of ascorbic acid 2‐glucoside (AA2G), a stabilized derivative of ascorbic acid, as an antioxidant during the thawing process following the direct freezing of mouse sperm at ‐80°C.

As, the experiments are based on the optimization of capacitation media adding 0.5 mM AA2G and on the comparison of its effects with the standard protocol that uses methyl‐beta‐cyclodextrin (MβCD) as cholesterol extractor [16]. Specifically, the latter is a chemically modified cyclodextrin, a cyclic sugar molecule derived from starch. It is widely used in biological and pharmaceutical applications due to its unique ability to interact with lipids and hydrophobic molecules. The combined effects of AA2G and MBCD were assessed in order to determine potential synergistic benefits. The assessment of fertility was conducted by the production of two‐cell embryos through in vitro fertilization (IVF), followed by blastocyst development.

## 2 Materials and Methods

### 2.1 Mice and Husbandry

Mice belonging to the strains C57BL/6NTacCnrm and C57BL/6JCnrm were bred at the Consiglio Nazionale delle Ricerche‐European Mouse Mutant Archive (CNR‐EMMA)‐Infrafrontier Specific Pathogen Free (SPF) barrier unit (Monterotondo Scalo, Rome, Italy). They were housed in individually ventilated cages (Tecniplast, Gazzada, Italy) at a temperature of 20°C ± 2°C, relative humidity of 55% ± 15% with 12–15 air changes per hour and a 12/12‐h light/dark cycle (7 a.m. to 7 p.m.). Certified dust‐free wood bedding (Scobis one, Mucedola, Settimo Milanese, Milan, Italy) was provided in the cages. Mice were fed a standardized mouse diet (4RFN and Emma 23, Mucedola, Italy) and were provided chlorinated, filtered water ad libitum. They were tested for microorganisms every 3 months using 6‐ to 8‐week‐old B6N sentinels. Serology was performed according to the FELASA recommendations [17].

### 2.2 Reagents

D‐(+)‐raffinose pentahydrate, *α*‐monothioglycerol (MTG), reduced L‐glutathione (GSH), bovine serum albumin (BSA, embryo tested), polyvinyl alcohol (PVA), methyl‐*β*‐cyclodextrin (MBCD), ascorbic acid 2‐Glucoside (AA2G), paraformaldehyde, Coomassie blue G250, and embryo‐tested water were purchased from Sigma–Aldrich (Merck KGaA, Darmstadt, Germany). Skimmed milk was sourced from BD Diagnostics (Le Pont de Claix, France) and human tubal fluid (HTF) medium from Millipore (Merck). Pregnant mare's serum gonadotropin (PMSG) and human chorionic gonadotropin (hCG) were purchased from Intervet (Milan, Italy). For sperm treatment, a modified Krebs–Ringer bicarbonate solution (TYH) containing 1.0 mg/mL PVA was prepared in‐house. The HTF medium was modified with the addition of CaCl_2_ to increase the Ca^2+^ concentration from 2.04 mM (regular concentration) to 5.14 mM (high concentration). Fluokit was purchased from Microptic (Barcelona, Spain).

### 2.3 Sperm Cryopreservation

Six males C57BL/6NTacCnrm (B6N) and six males C57BL/ 6JCnrm (B6J) in total were used for this experiment.

According to EMMA procedures [18], the *caudae epididymides* and the *vasa deferentia* of two 3‐month‐old males were placed in 2 mL of the cryoprotective medium (CPM), consisting of 18% w/v raffinose, 3% w/v skim milk, and 477 µM MTG. Spermatozoa were allowed to disperse from the tissues for 10 min at 37°C. The pooled sperm were loaded into 0.25 mL French straws (IMV Technologies, France), and frozen directly at ‐80° freezer as previously reported [14]. This procedure was repeated 3 times to produce three different sperm samples.

### 2.4 Sperm Thawing and Treatment

Frozen spermatozoa were thawed by placing the straws in a water bath at 37°C for 8 min.

For the sperm treatment, after thawing, 30 µL of spermatozoa were transferred into a capacitation drop consisting of 90 µL of TYH with 0.75 mM MBCD (M) or 0.75 mM MBCD associated with the 0.5 mM AA2G (MA). Spermatozoa were incubated for 30 min at 37°C (5% CO_2_) before each analysis of motility, vitality, acrosome reaction, and IVF.

### 2.5 Sperm Motility and Viability

After the treatment, sperm motility and viability were assessed using the Sperm Class Analyzer (SCA; Microptic, Barcelona, Spain) with SCA Research 6.5 software version, configured for mouse parameters. Motility analyses were conducted on 20 µm‐deep counting chamber SCA slides (Microptic). Each chamber was loaded with a 4 µL semen sample.

Sperm tracks were captured at 37°C using a 10× negative‐phase contrast objective.

Sperm cells were considered progressive if VAP *>* 50 µm/s and STR *>* 50% and were classified as rapid if the linear velocity was *>* 25 µm/s as previously reported in Raspa et al. [16].

Sperm viability was assessed using the FluoVit kit (Microptic), following the manufacturer's guidelines. Briefly, after treatment, 10 µL of semen samples were mixed with 1 µL of BLUE solution (Hoechst 33342 and trihydrochloride trihydrate) and incubated for 5 min at 37°C. Then, 1 µL of RED solution (Propidium Iodide) was added for 1 min. Six microliters of stained samples were mounted with mounting medium Eukitt (O. Kindler, Orsatec GmbH, Bobingen, Germany) and analyzed using a long bandpass filter (EX 333–380, EM 420, DM 440). Alive spermatozoa were stained blue, while dead spermatozoa were stained red.

For each sample and analysis, a minimum of 500 spermatozoa were examined, and the analyses were repeated for each batch. The proportion of viable sperm in each specimen was calculated as the average (across the three replicates) of the total number of live spermatozoa, divided by the total number of counted spermatozoa.

### 2.6 Acrosome Reaction

Coomassie blue staining was conducted following the method previously described [19] with a slight modification as documented in an earlier report [20]

Thawed spermatozoa, after treatment, were fixed in a 4% paraformaldehyde solution in PBS for 10 min and then washed with 0.1 M ammonium acetate at pH 9.0 using a brief centrifugation step (1500× *g* for 2 min). The samples were re‐suspended in PBS and spread onto slides for air drying. The sperm smear was subsequently stained with a solution of 0.22% Coomassie blue G250 in 50% methanol and 10% glacial acetic acid for 2 min. After staining, the slides were washed 4–5 times with distilled H_2_O and mounted.

Spermatozoa were examined under bright field microscopy (Nikon) with a 40× objective. Spermatozoa exhibiting a dark blue band over the acrosome region were considered to have an intact acrosome, while those lacking the blue band were categorized as AR. The percentage of acrosome‐reacted spermatozoa (AR%) was determined by assessing at least 200 spermatozoa on each slide.

### 2.7 In Vitro Fertilization and Embryo Development

IVF was conducted following the protocol outlined by Nakagata et al. [21], with some modifications as detailed in a previous study [20, 21].

Cryopreserved spermatozoa were thawed in a 37°C water bath and treated, as previously described, for 30 min. Twelve 4‐week‐old B6N or B6J females were previously superovulated through an intraperitoneal injection of 5 IU PMSG, followed by an injection of 5 IU hCG 48 h later. At 12–14 h post‐hCG injection, the females were euthanized through cervical dislocation. Their oviducts were then extracted, and four cumulus–oocyte complexes (COCs) were placed into a 250 µL fertilization drop containing 250 µL of HTF medium and 1 mM reduced L‐glutathione (GSH). The COCs were allowed to incubate for 20 min. After treatment, 20 µL of sperm suspension (at a final concentration of 1 × 10^5^ cells/mL) was collected and used to inseminate the COCs. After co‐incubation for 4 h, the presumptive zygotes were washed 3 times in 200 µL of the HTF medium and then cultured overnight. After approximately 18 h, 2‐cell embryos were harvested. The IVF rate, expressed as the percentage of 2‐cell embryos relative to the number of co‐incubated oocytes, was determined. For each treatment, three IVFs were carried out, one for each sperm sample, using in total 36 females.

For quality control, some of the 2‐cell embryos were assessed by the in vitro embryo culture in the potassium simplex‐optimized medium supplemented with amino acids (KSOM^AA^) [22] for 72 h until the blastocyst stage. For each group, 30 embryos were cultured and three replications were performed. Results are expressed as the percentage of blastocysts developing from the number of 2‐cell embryos cultured.

## 3 Results

### 3.1 Sperm Motility and Vitality

Qualitative analysis was performed on 3 samples per wildtype at different times: 1, 6, and 12 months.

The evaluation of mobility did not yield significant differences comparing the control group (M) and the treated group (MA) on both B6J and B6N spermatozoa. Overall, a decrease in the percentage of total and progressive spermatozoa was observed, either in control and treated for both lines, from 1 to 12 months. Sperm velocity parameters were also unaffected.

**FIGURE 1:**
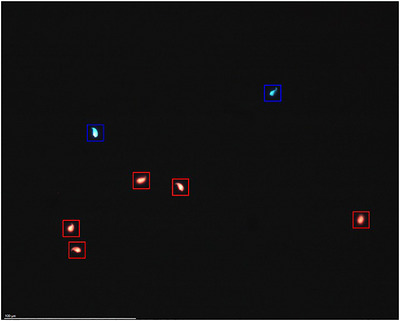
SCA system images (B6N). Explanatory images of viability analysis by SCA system of wildtype murine spermatozoa. The blue fluorescence represents live sperms, and the red represents dead ones.

**FIGURE 2:**
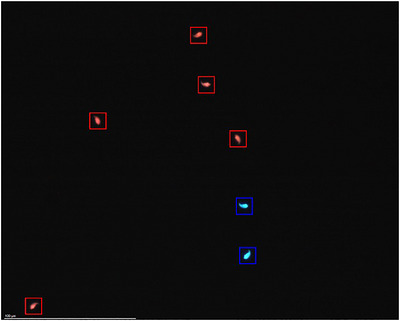
SCA system images (B6J). Explanatory images of viability analysis by SCA system of wildtype murine spermatozoa. The blue fluorescence represents live sperms, and the red represents dead ones.

The proportions of living (blue) and dead (red) spermatozoa were determined using the SCA Analyzer (Microptic), as depicted in Figures 1 and 2, respectively for B6N and B6J.

In B6J, in general the average percentage viability measured over months is lower than in B6N in both control and treated.

**FIGURE 3:**
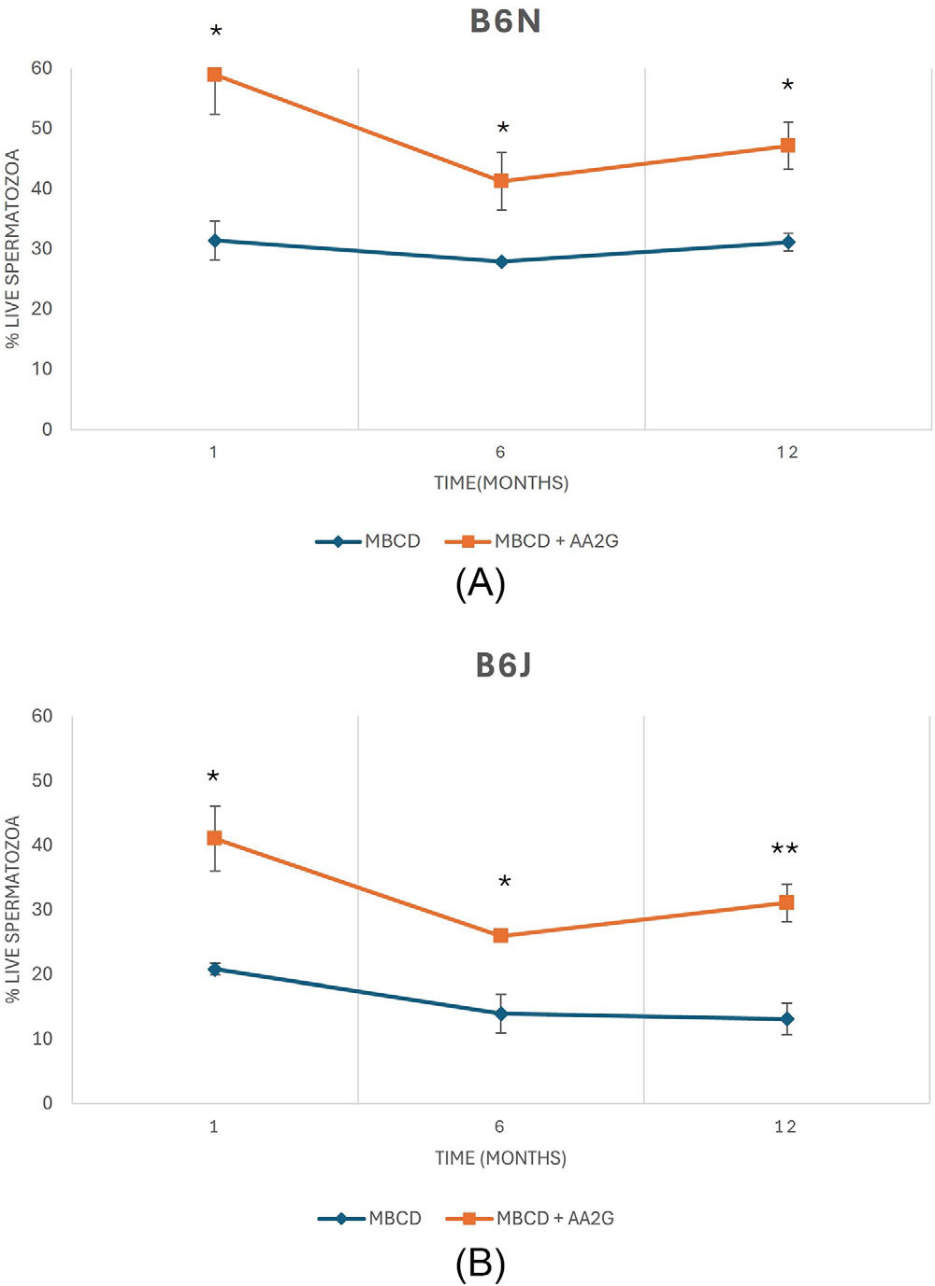
Sperm viability analysis. Viability of B6N and B6J spermatozoa frozen directly at ‐80°C. In blue, the trend of the mean viability of the control samples over the three times and in orange the tendency of the treated samples with the corresponding standard deviations and significance (*n* = 3, * p<0.05; **p<0.01).

After 1 month of freezing at ‐80°C, for B6N, the percentage of live spermatozoa following treatment with MBCD was 31.38% ± 3.23%. This value increased significantly to 58.93% ± 6.58% (p < 0.05) after MA treatment (Figure 3A). On the other hand, for B6J we started with a percentage of 20.77% ± 0.91% in the control and went up to 41.00%± 5.03% (p < 0.05) in the AA2G (Figure 3B).

After 6 months, this percentage for B6N is maintained in the control (M) with a value of 27.87% ± 0.42%. and in the MA treatment there is still an increase, however smaller, to a percentage of 41.25% ± 4.78% (p < 0.05). For B6J, the live sperm count with MBCD significantly dropped, as compared to sample stored for 1 month (13.9% ± 3.00% vs 20.77% ± 0.91%) and after antioxidant treatment was increased to 25.9%± 0.9% (p < 0.05).

Finally, after 12 months, the percentage in B6N fell from 31.11% ± 1.48% in the M control to 47.14% ± 3.94% in the MA treatment. In B6J live spermatozoa increased from 13.11%± 2.40% with MBCD to 31.04%± 2.91% (p< 0.001) with the synergy of MBCD and AA2G.

### 3.2 Acrosome Reaction

**FIGURE 4:**
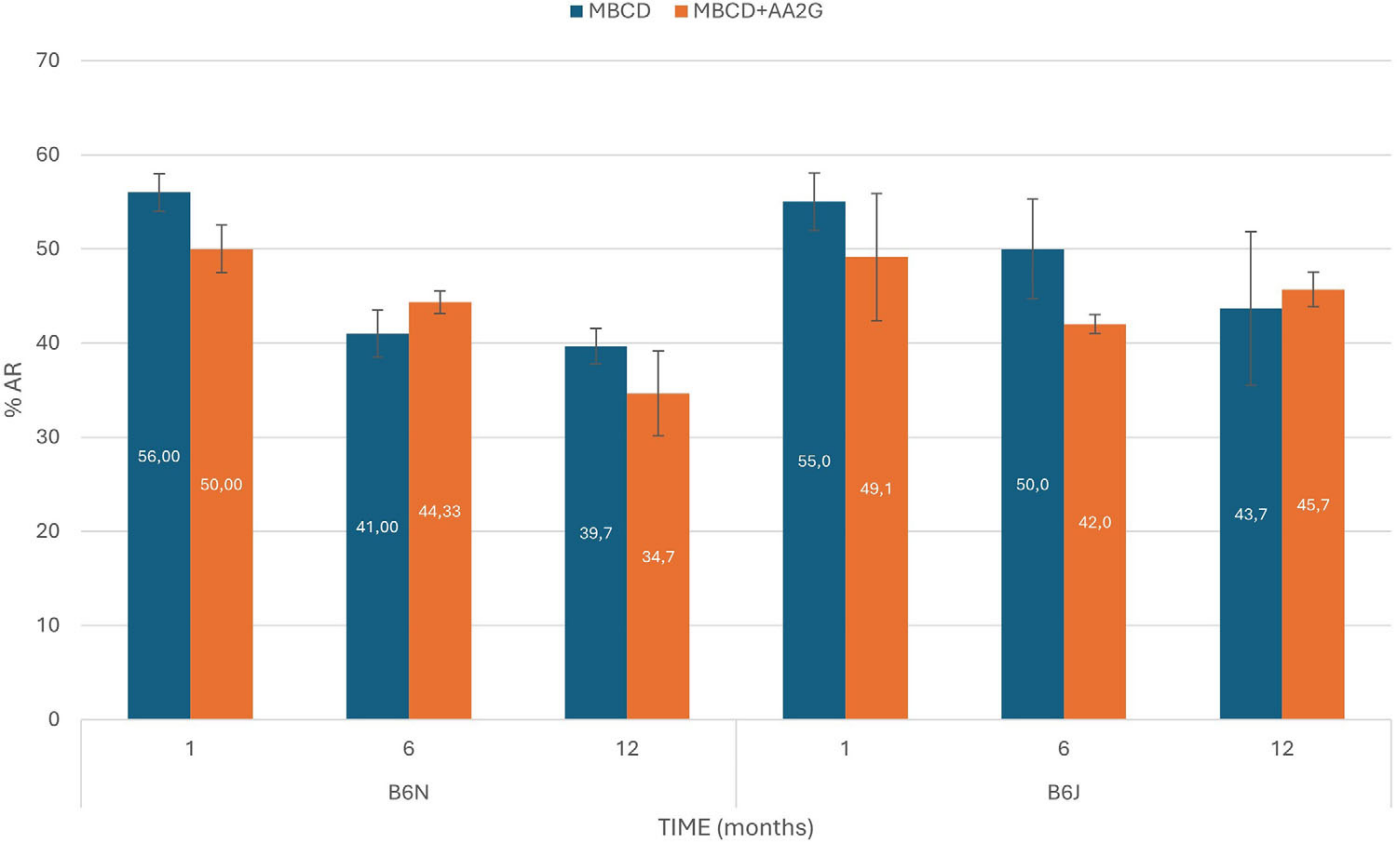
Acrosome reaction analyses. Percentage of acrosome‐reacted spermatozoa in B6N and B6J males, after different treatments (*n* = 3, p > 0.05).

The qualitative analysis of the AR state was performed after 30 min of capacitation in MBCD and in MBCD with the addition of AA2G. No significant difference was observed in the percentage of reacted spermatozoa between control and treated (p > 0.05). In any case, in both M and MA, the percentages were around 50% and decreased slightly from 1 to 12 months. These results also showed (Figure 4) no differences in relation to the murine sperm background (B6N and B6J).

### 3.3 In Vitro Fertilization and Embryo Development

**FIGURE 5:**
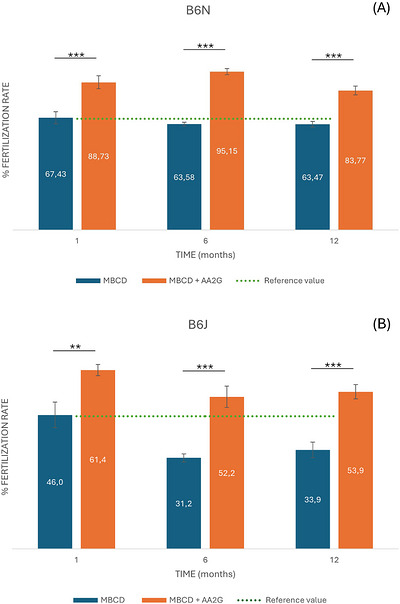
(A and B) Impact of treatment on in vitro fertilization. The in vitro fertilization success rate was evaluated using cryopreserved B6N (A) and B6J (B) spermatozoa directly at ‐80°C that were treated with MBCD (M) or a combination of MBCD and AA2G (MA) for 30 min at thawing phase. Each experimental group was tested in triplicate (*n* = 3, **p < 0.01; ***p < 0.001).

The number of oocytes collected and the number of 2‐cell embryos obtained were analyzed to assess the effect of sperm treatment on IVF. A significant improvement in the B6N fertilization was achieved by treated spermatozoa (MA) compared to the M group (67.43% ± 3.6% vs. 88.7% ± 3.9%, p < 0.001 at 1 month; 63.6% ± 1.1% vs 95.2% ± 1.8%, p < 0.001 at 6 months and finally 63.5% ± 1.7% vs. 83.8% ± 1.7%, p < 0.001 at 12 months) as described in Figure 5A.

In B6J (Figure 5B), there was also a significant increase in fertilization rate after MA treatment. Fertilizing capacity increased from 46% ± 4.4% with MBCD to 61.4% ± 1.9% in the AA2G‐treated sample at 1 month (p < 0.01), from 31.2% ± 1.4% to 52.2% ± 3.6% at 6 months (p < 0.001) and from 33.9% ± 2.7% to 52.9% ± 2.4% at 12 months (p < 0.001).

Notably, in the M group, the percentage of 2‐cells obtained was significantly reduced after 6 months compared to 1 month (31.2% ± 1.4% vs. 46% ± 4.4% p < 0.05) as described [23], interestingly the MA treatment brought the fertilization rate back to values close to the sample frozen for 1 month (reference value)

The use of the antioxidant was also evaluated on the basis of the number of embryos that had reached the blastocyst stage. In B6N and B6J (Table 1), AA2G had no effect on blastocyst development in vitro [16] and the percentages of blastocysts obtained ranged between 75% and 80% in both control and treated and regardless of timing (p > 0.05).

**TABLE 1:** Embryo development. Blastocyst development of embryos derived from spermatozoa treated for 30 min with MBCD and AA2G (MA) at time 1, 6, and 12 months.

% Blastocysts			
	1 month	6 months	12 months
B6N	88.3% ± 1.7%	86.7% ± 1.7%	90% ± 5.8%
B6J	83.3% ± 3.3%	81.7% ± 6%	86,7% ± 4.4%

## 4 Discussion

Mouse repositories play a crucial role in ensuring the cryopreservation and distribution of mouse models for the scientific community [14]. Traditionally, embryo freezing in liquid nitrogen has been the preferred method for archiving and transporting mouse lines. However, over the past decade, sperm freezing has emerged as a more cost‐effective and practical alternative [7].

Previous studies have shown that frozen sperm can be stored for long periods of up to 5 years in a freezer at ‐80°C [11]. Furthermore, spermatozoa from both wild‐type and genetically altered (GA) lines remained fertile even after being transferred to liquid nitrogen (LN_2_).

The use of cryopreservation protocols, while essential, can result in cellular damage that undermines both functionality and fertilizing potential [24]. In particular, the plasma membrane is considered the primary site of damage during this process. Alterations in male gametes due to cryopreservation are closely linked to the properties of the sperm membrane, including its phospholipid composition, cholesterol content, permeability, and osmotic tolerance [25]. A key challenge is the osmotic stress spermatozoa endure during cooling [24], which can lead to structural and functional disruptions. Specifically, membrane phase transitions induced by cooling can modify the structure and conformation of biomolecules (such as lipids and proteins), potentially in an irreversible manner, thus impairing membrane function [25]. The reduction in membrane fluidity associated with cooling can facilitate the loss of intracellular solutes and trigger irreversible conformational changes. Additionally, the formation of extracellular ice increases the concentration of external solutes, promoting cell dehydration through the outward movement of water along the osmotic gradient. Cells have defined osmotic tolerance limits, beyond which apoptosis is induced [24].

However, by considering temperature and the action of cryoprotectants, it is possible to modulate membrane phase behaviour at subzero temperatures [10]. Cryoprotectants not only regulate the timing and dynamics of ice formation and the cellular osmotic response but also stabilize proteins and membranes [25]. Increasing the glass transition temperature could be beneficial for handling cryopreserved samples under suboptimal conditions and may enable storage at higher temperatures, such as ‐80°C, instead of liquid nitrogen [11].

In order to optimize critical steps while preventing damage to nuclear DNA, epigenetic patterns and gene expression, and ultimately to the developmental competence and health of the offspring, it is important to investigate the use of antioxidants [16] in cryopreservation and thawing protocols.

The function of antioxidants, such as ascorbic acid, in relation to sperm quality and fertility is complex and dependent on a delicate balance between the production of reactive oxygen species (ROS) and the antioxidant defense system. Elevated antioxidant levels have been shown to exert a favorable influence on sperm characteristics and function; however, excessive concentrations may exert a deleterious effect on the activity of antioxidant enzymes [26].

We previously tested AA2G in combination with MBCD, a compound routinely used to enhance sperm capacitation in standard fertilization assays. We used B6N and B6J substrains, as these genetic backgrounds are the most represented in our biobank and reported that AA2G treatment enhanced survival, quality, and functionality of B6N and B6J sperm frozen in liquid nitrogen [16].

Ascorbic acid supplementation during cryopreservation has been shown to improve sperm motility, viability, DNA integrity, and mitochondrial function, reducing ROS production and protecting human sperm from cryodamage [27, 28, 29].

It was also reported an increased progressive sperm motility in other species including Awassi ram [30] buffalo [31] and rooster [32]. However, the effects of supplementation with ascorbic acid may be dependent on the concentration.

On the other hand, some studies have not reported beneficial effects of ascorbic acid on post‐thawing sperm parameters in sperm of some animal species [33, 34] when used in freeze extenders or after thawing.

These inconsistencies may stem from variations in concentration, methodology, procedural differences, or the susceptibility of ascorbic acid to thermal and oxidative degradation. To overcome these limitations, this study used AA2G, a more stable derivative of ascorbic acid [16] and a potent antioxidant agent with both in vitro and in vivo efficacy [35, 36, 37].

Storing sperm at ‐80°C offers several advantages over LN_2_. It eliminates the need for dedicated storage space, is more cost‐effective since it does not require constant refilling like LN_2_ tanks and only requires electrical power. In addition, most small laboratories already have access to freezers at ‐80°C. Based on these results, the use of the ‐80° freezer was extended to the freezing procedure. We have demonstrated that the entire process could be performed without LN_2_ using B6N spermatozoa [14].

Previous studies have shown that frozen sperm can be stored for long periods of up to 5 years in a freezer at ‐80°C [11]. Furthermore, spermatozoa from both wild‐type and genetically altered (GA) lines remained fertile even after being transferred to liquid nitrogen (LN_2_). We know from a recent study that cryopreservation at ‐80°C significantly increased ultrastructure damage compared to LN_2_. Particularly, the survival and fertility of mouse spermatozoa frozen and maintained at ‐80°C was strain dependent and that B6J is more sensitive than B6N [23].

In this work we used AA2G, supplemented to the capacitation medium, to evaluate if the use of this antioxidant could be advantageous for spermatozoa frozen and maintained at ‐80°C and counteract the negative effects observed.

We used a combination of AA2G and MBCD at thawing after 1, 6, and 12 months of storage in a freezer at ‐80°C. This treatment significantly increased sperm vitality in both B6N and B6J males. These improvements correlated with a notable rise in fertilization rates. Additionally, AA2G treatment had no impact on the quality of 2‐cell embryos, which developed normally to the blastocyst stage.

B6J sperm were more sensitive to cryopreservation‐induced damage, particularly to the head and tail, reducing their fertilizing capacity compared to other strains [38, 39]. After thawing, B6J spermatozoa generally showed lower survival and fertilizing capacity than B6N. However, treatment with AA2G significantly improves both parameters in both strains compared to MBCD alone.

MBCD was essential for significant improvements in capacitation and fertility, as it facilitates cholesterol efflux, a key step for enhancing the fertilization potential of frozen/thawed sperm [40]. When combined with MBCD, AA2G improved sperm survival in both strains, directly increasing IVF success.

The role of antioxidants like ascorbic acid in sperm quality is complex, balancing ROS generation and antioxidant defenses. While optimized antioxidant levels benefit sperm function, excessive concentrations may impair enzyme activity [26].

Incorporating AA2G with MBCD in the capacitation medium enhances the efficiency of sperm thawing, storage, and use in research, reducing the number of animals needed aligning with the 3Rs principles in animal research. Further studies on AA2G supplementation during freezing and its effects across genetic backgrounds and engineered mouse models will be key to refining cryopreservation protocols [16].

## 5 Conclusion

In conclusion, these results may help to design new protocols or optimize those already validated by promoting the use of antioxidants in freezing at ‐80° as they have a significant effect on reducing oxidative stress, making spermatozoa qualitatively comparable to freezing in LN_2_ [11].

## Author Contributions

**Conceptualization**: Ferdinando Scavizzi and Marcello Raspa. **Formal analysis**: Alessia Paradiso and Renata Paoletti. **Investigation**: Alessia Paradiso and Renata Paoletti. **Methodology**: Alessia Paradiso and Nicola Bernabò. **Project administration**: Marcello Raspa and Ferdinando Scavizzi. **Supervision**: Ferdinando Scavizzi, Barbara Barboni, and Marcello Raspa. **Visualization**: Simone Cassetti and Nicola Bernabò. **Writing – original draft**: Ferdinando Scavizzi, Marcello Raspa, Barbara Barboni, and Nicola Bernabò. All authors have read and agreed to the published version of the manuscript.

## Funding

This project is funded by MUR DM352/2022, PNRR: mission 4, component 2 (“From research to company”).

## Ethics Statement

The animal study protocol was approved by the Institutional Review Board (Organismo Preposto al Benessere degli Animali, OPBA) of the Institute of Biochemistry and Cell Biology EMMA/Infrafrontier (Protocol number 0000079 of January 18, 2016) for studies involving animals.

## Conflicts of Interest

The authors declare no conflicts of interest.

## Data Availability

All data generated or analyzed during this study are included in this published paper.

## References

[1] A. Bradley , K. Anastassiadis , A. Ayadi , et al., “The Mammalian Gene Function Resource: The international knockout mouse consortium,” Mammalian Genome 23, no. 9‐10 (2012): 580–586, 10.1007/s00335-012-9422-2. 22968824 PMC3463800

[2] K. C. K. Lloyd , “A Knockout Mouse Resource for the Biomedical Research Community,” Annals of the New York Academy of Sciences 1245, no. 1 (2011): 24–26, 10.1111/j.1749-6632.2011.06311.x. 22211970 PMC4070945

[3] R. Taft , M. Davisson , and M. Wiles , “Know Thy Mouse,” Trends in Genetics 22, no. 12 (2006): 649–653, 10.1016/j.tig.2006.09.010. 17007958

[4] M. Hrabě de Angelis , G. Nicholson , and M. Selloum , “Analysis of Mammalian Gene Function Through Broad‐Based Phenotypic Screens Across a Consortium of Mouse Clinics,” Nature Genetics 47, no. 9 (2015): 969–978, 10.1038/ng.3360. 26214591 PMC4564951

[5] T. F. Meehan , N. Conte , D. B. West , et al., “Disease Model Discovery From 3,328 Gene Knockouts by The International Mouse Phenotyping Consortium,” Nature Genetics 49, no. 8 (2017): 1231–1238, 10.1038/ng.3901. 28650483 PMC5546242

[6] H. Wang , H. Yang , C. S. Shivalila , et al., “One‐Step Generation of Mice Carrying Mutations in Multiple Genes by CRISPR/Cas‐Mediated Genome Engineering,” Cell 153, no. 4 (2013): 910–918, 10.1016/j.cell.2013.04.025. 23643243 PMC3969854

[7] M. Raspa , M. Fray , R. Paoletti , L. Montoliu , A. Giuliani , and F. Scavizzi , “Long Term Maintenance of Frozen Mouse Spermatozoa at −80°C,” Theriogenology 107 (2018): 41–49, 10.1016/j.theriogenology.2017.10.036. 29128700

[8] D. G. Whittingham , S. P. Leibo , and P. Mazur , “Survival of Mouse Embryos Frozen to ‐196 Degrees and ‐269 Degrees C,” Science 178, no. 4059 (1972): 411–414. 5077328

[9] I. Wilmut , “The Low Temperature Preservation of Mammalian Embryos,” Reproduction (Cambridge, England) 31, no. 3 (1972): 513–514, 10.1530/jrf.0.0310513. 4120076

[10] I. Wilmut , “The Effect of Cooling Rate, Warming Rate, Cryoprotective Agent and Stage of Development of Survival of Mouse Embryos During Freezing and Thawing,” Life Sciences 11, no. 22 (1972): 1071–1079, 10.1016/0024-3205(72)90215-9. 4663808

[11] M. Raspa , S. Putti , R. Paoletti , et al., “The Impact of Five Years Storage/Biobanking at −80°C on Mouse Spermatozoa Fertility, Physiology, and Function,” Andrology 9, no. 3 (2021): 989–999, 10.1111/andr.12971. 33427410

[12] N. Nakagata , “Cryopreservation of Mouse Spermatozoa,” Mammalian Genome 11, no. 7 (2000): 572–576, 10.1007/s003350010109. 10886025

[13] J. M. Sztein , K. Noble , J. S. Farley , and L. E. Mobraaten , “Comparison of Permeating and Nonpermeating Cryoprotectants for Mouse Sperm Cryopreservation,” Cryobiology 42, no. 1 (2001): 28–39, 10.1006/cryo.2001.2300. 11336487

[14] M. Raspa , M. Fray , R. Paoletti , L. Montoliu , A. Giuliani , and F. Scavizzi , “A New, Simple and Efficient Liquid Nitrogen Free Method to Cryopreserve Mouse Spermatozoa at −80 °C,” Theriogenology 119 (2018): 52–59, 10.1016/j.theriogenology.2018.06.020. 29982136

[15] M. Raspa , M. Guan , R. Paoletti , et al., “Dry Ice Is a Reliable Substrate for the Distribution of Frozen Mouse Spermatozoa: A Multi‐centric Study,” Theriogenology 96 (2017): 49–57, 10.1016/j.theriogenology.2017.04.003. 28532839

[16] M. Raspa , R. Paoletti , and F. Scavizzi , “Ascorbic Acid 2‐Glucoside Improves Survival, Quality, and Fertility of Frozen‐Thawed C57Bl/6J and C57Bl/6N Mouse Spermatozoa,” Andrology 13, no. 6 (2025): 1601–1614, 10.1111/andr.13768, Published online September 27, 2024. 39330618 PMC12368942

[17] M. Mähler Convenor and M. Berard , “FELASA Recommendations for the Health Monitoring of Mouse, Rat, Hamster, guinea Pig and Rabbit Colonies in Breeding and Experimental Units,” Laboratory Animals 48, no. 3 (2014): 178–192, 10.1177/0023677213516312. 24496575

[18] G. C. Ostermeier , M. V. Wiles , J. S. Farley , and R. A. Taft , “Conserving, Distributing and Managing Genetically Modified Mouse Lines by Sperm Cryopreservation,” PLoS ONE 3, no. 7 (2008): e2792, 10.1371/journal.pone.0002792. 18665210 PMC2453316

[19] J. L. Larson and D. J. Miller , “Simple Histochemical Stain for Acrosomes on Sperm From Several Species,” Molecular Reproduction and Development 52, no. 4 (1999): 445–449, 10.1002/(SICI)1098-2795(199904)52:4<445::AID-MRD14>3.0.CO;2-6. 10092125

[20] M. Raspa , R. Paoletti , E. Mahabir , and F. Scavizzi , “D‐Aspartate Treatment in Vitro Improves Mouse Sperm Fertility in Young B6N Mice,” Theriogenology 148 (2020): 60–67, 10.1016/j.theriogenology.2020.02.031. 32142981

[21] N. Nakagata , T. Takeo , K. Fukumoto , et al., “Rescue in Vitro Fertilization Method for Legacy Stock of Frozen Mouse Sperm,” Journal of Reproduction and Development 60, no. 2 (2014): 168–171, 10.1262/jrd.2013-141. 24492659 PMC3999397

[22] N. Bernabò , L. Valbonetti , M. Raspa , et al., “Graphene Oxide Improves in Vitro Fertilization in Mice with no Impact on Embryo Development and Preserves the Membrane Microdomains Architecture,” Frontiers in Bioengineering and Biotechnology 8 (2020): 629, 10.3389/fbioe.2020.00629. 32612987 PMC7308453

[23] M. Peltier , M. Raspa , S. Putti , R. Paoletti , F. Scavizzi , and E. Mahabir , “Cryopreservation at −80°C Impacts Sperm Integrity and Fertility in a Mouse Strain‐Dependent Manner,” Theriogenology 245 (2025): 117523, 10.1016/j.theriogenology.2025.117523. 40513374

[24] A. Agarwal , T. M. Said , M. A. Bedaiwy , J. Banerjee , and J. G. Alvarez , “Oxidative Stress in an Assisted Reproductive Techniques Setting,” Fertility and Sterility 86, no. 3 (2006): 503–512, 10.1016/j.fertnstert.2006.02.088. 16860798

[25] W. V. Holt , A. Medrano , L. M. Thurston , and P. F. Watson , “The Significance of Cooling Rates and Animal Variability for Boar Sperm Cryopreservation: Insights From the Cryomicroscope,” Theriogenology 63, no. 2 (2005): 370–382, 10.1016/j.theriogenology.2004.09.018. 15626405

[26] J. H. Hu , W. Q. Tian , X. L. Zhao , et al., “The Cryoprotective Effects of Ascorbic Acid Supplementation on Bovine Semen Quality,” Animal Reproduction Science 121, no. 1‐2 (2010): 72–77, 10.1016/j.anireprosci.2010.04.180. 20478670

[27] Z. Li , Q. Lin , R. Liu , W. Xiao , and W. Liu , “Protective Effects of Ascorbate and Catalase on Human Spermatozoa during Cryopreservation,” Journal of Andrology 31, no. 5 (2010): 437–444, 10.2164/jandrol.109.007849. 19834132

[28] C. S. Branco , M. E. Garcez , F. F. Pasqualotto , B. Erdtman , and M. Salvador , “Resveratrol and Ascorbic Acid Prevent DNA Damage Induced by Cryopreservation in human Semen,” Cryobiology 60, no. 2 (2010): 235–237, 10.1016/j.cryobiol.2009.10.012. 19895799

[29] H. Fanaei , S. Khayat , I. Halvaei , et al., “Effects of Ascorbic Acid on Sperm Motility, Viability, Acrosome Reaction and DNA Integrity in Teratozoospermic Samples,” Iranian Journal of Reproductive Medicine 12, no. 2 (2014): 103–110. 24799867 PMC4009562

[30] O. I. Azawi and E. K. Hussein , “Effect of Vitamins C or E Supplementation to Tris Diluent on the Semen Quality of Awassi Rams Preserved at 5 °C,” Veterinary Research Forum 4, no. 3 (2013): 157–160. 25653790 PMC4312374

[31] V. S. Raina , A. K. Gupta , and K. Singh , “Effect of Antioxidant Fortification on Preservability of Buffalo Semen,” Asian‐Australasian Journal of Animal Sciences 15, no. 1 (2002): 16–18, 10.5713/ajas.2002.16.

[32] M. R. Amini , H. Kohram , A. Zare Shahaneh , M. Zhandi , H. Sharideh , and M. M. Nabi , “The Effects of Different Levels of Vitamin E and Vitamin C in Modified Beltsville Extender on Rooster Post‐Thawed Sperm Quality,” Cell and Tissue Banking 16, no. 4 (2015): 587–592, 10.1007/s10561-015-9506-9. 25779925

[33] M. R. Fernández‐Santos , F. Martínez‐Pastor , V. García‐Macías , et al., “Sperm Characteristics and DNA Integrity of Iberian Red Deer (*Cervus elaphus hispanicus*) Epididymal Spermatozoa Frozen in the Presence of Enzymatic and Nonenzymatic Antioxidants,” Journal of Andrology 28, no. 2 (2007): 294–305, 10.2164/jandrol.106.000935. 17079744

[34] M. Ivanova , D. Abadjieva , D. Gradinarska , et al., “Post Thaw Treatment of Frozen Buffalo Semen With Antioxidants Vitamin C and 2‐Mercaptoethanol,” Biotechnology & Biotechnological Equipment 34, no. 1 (2020): 1315–1322, 10.1080/13102818.2020.1837013.

[35] W. Y. Huang , P. C. Lee , L. K. Huang , L. P. Lu , and W. C. Liao , “Stability Studies of Ascorbic Acid 2‐Glucoside in Cosmetic Lotion Using Surface Response Methodology,” Bioorganic & Medicinal Chemistry Letters 23, no. 6 (2013): 1583–1587, 10.1016/j.bmcl.2013.01.111. 23416010

[36] I. Yamamoto and N. Muto , “Bioavailability and Biological Activity of L‐Ascorbic Acid 2‐O‐α‐Glucoside,” Journal of Nutritional Science and Vitaminology 38, no. Special (1992): 161–164, 10.3177/jnsv.38.Special_161. 1297731

[37] C. Jacques , C. Genies , D. Bacqueville , et al., “Ascorbic Acid 2‐Glucoside: An Ascorbic Acid Pro‐Drug With Longer‐Term Antioxidant Efficacy in Skin,” International Journal of Cosmetic Science 43, no. 6 (2021): 691–702, 10.1111/ics.12745. 34679221

[38] J. M. Sztein , J. S. Farley , and L. E. Mobraaten , “In Vitro Fertilization With Cryopreserved Inbred Mouse Sperm1,” Biology of Reproduction 63, no. 6 (2000): 1774–1780, 10.1095/biolreprod63.6.1774. 11090448

[39] H. Nishizono , M. Shioda , T. Takeo , T. Irie , and N. Nakagata , “Decrease of Fertilizing Ability of Mouse Spermatozoa After Freezing and Thawing Is Related to Cellular Injury1,” Biology of Reproduction 71, no. 3 (2004): 973–978, 10.1095/biolreprod.103.024422. 15151925

[40] T. Takeo , T. Hoshii , Y. Kondo , et al., “Methyl‐Beta‐Cyclodextrin Improves Fertilizing Ability of C57BL/6 Mouse Sperm After Freezing and Thawing by Facilitating Cholesterol Efflux From the Cells1,” Biology of Reproduction 78, no. 3 (2008): 546–551, 10.1095/biolreprod.107.065359. 18046011

